# An Alginate Hydrogel–Lipid Nanodispersion Bio-Mask: A Preliminary Study of Skin Hydration, Barrier Function, and Regenerative Potential

**DOI:** 10.3390/ma19102108

**Published:** 2026-05-17

**Authors:** Małgorzata Miastkowska, Agnieszka Kulawik-Pióro, Anna Sienkiewicz, Anna Łętocha, Katarzyna Malarz, Anna Mrozek-Wilczkiewicz, Katarzyna Bialik-Wąs

**Affiliations:** 1 Cracow University of Technology, Faculty of Chemical Engineering and Technology, Warszawska 24, 31-155 Cracow, Poland; agnieszka.kulawik-pioro@pk.edu.pl (A.K.-P.); anna.sienkiewicz@pk.edu.pl (A.S.); anna.letocha@pk.edu.pl (A.Ł.); katarzyna.bialik-was@pk.edu.pl (K.B.-W.); 2 August Chelkowski Institute of Physics, Faculty of Science and Technology, University of Silesia in Katowice, 75 Pułku Piechoty 1A, 41-500 Chorzow, Poland; katarzyna.malarz@us.edu.pl (K.M.); anna.mrozek-wilczkiewicz@us.edu.pl (A.M.-W.); 3 Department of Systems Biology and Engineering, Faculty of Automatic Control, Electronics and Computer Science, Silesian University of Technology, Akademicka 16, 44-100 Gliwice, Poland

**Keywords:** alginate hydrogel, lipid nanodispersion, bio-mask, skin hydration, transepidermal water loss (TEWL), skin barrier, procollagen synthesis

## Abstract

Laser therapy is commonly associated with transient skin reactions such as erythema and edema, creating a need for effective post-procedural skincare strategies. In this study, we developed and characterized a novel bio-mask that integrates a hydrogel matrix with a lipid nanodispersion system designed to simultaneously deliver hydrophilic and hydrophobic active compounds. The key innovation of this formulation lies in the combination of a highly hydrophilic hydrogel structure with lipid nanoparticles embedded within a polymeric network, enabling enhanced bioavailability of active ingredients. Preliminary observations from instrumental measurements in a small group of healthy volunteers suggest that a single 60 min application resulted in notable improvements in skin hydration and elasticity, along with a reduction in transepidermal water loss (TEWL), erythema, and skin sensitivity. Furthermore, both the complete formulation and its individual components exhibited inhibitory activity against collagen and elastin glycation, while promoting type I procollagen synthesis. Importantly, this study provides new evidence for the synergistic interaction between hydrogel matrices and lipid nanodispersion systems in modulating skin barrier function and biochemical aging markers. The formulation, composed entirely of ingredients of natural origin, proved to be an effective carrier for active compounds and showed measurable benefits for skin hydration and barrier-related parameters.

## 1. Introduction

The integration of laser technology into medical practice has expanded significantly, becoming a widely adopted tool across multiple specialties, including dentistry, dermatology, ophthalmology, and surgery. They have replaced the traditional treatment method and become safe and effective [1,2]. Fractional CO_2_ lasers are widely used in esthetic medicine [1,3] to create small microcolumns, i.e., controlled damage, in the dermis. As a result, evaporation occurs and the tissue coagulates in microthermal treatment zones. Because the healing process is activated, the skin produces more collagen and elastin after the treatment [4]. The skin becomes revitalized, and it is characterized by improved firmness and tone, reduced fine wrinkles, and smoothed furrows. In addition, laser therapy is used to remove scars after acne, surgeries, stretch marks, vascular discoloration, and pigmentation. The fractional laser treatment is considered minimally invasive; however, if no proper post-treatment care is applied, side effects may occur, including post-inflammatory hyperpigmentation [3,5,6].

Currently, post-laser care typically involves the use of multiple formulations with distinct functions, including cooling masks providing immediate hydration and thermal relief, as well as separate topical preparations intended to support longer-term skin conditioning and recovery [7,8,9,10,11,12,13,14].

However, most available systems are designed to fulfill either immediate physical functions (e.g., cooling and hydration) or longer-term biochemical activity related to skin conditioning. The integration of these functionalities into a single material system remains limited [15]. This gap highlights the need for advanced biomaterial platforms capable of combining both immediate and sustained effects within a unified structure.

Hydrogel-based systems are particularly suitable for such applications due to their ability to form an occlusive layer, maintain a moist microenvironment, and provide immediate cooling and soothing effects [16]. Despite these advantages, conventional hydrogel systems are primarily suitable for the incorporation and release of hydrophilic compounds, whereas the effective delivery of lipophilic bioactive substances remains limited [17]. To overcome this challenge, hybrid systems combining hydrogels with lipid-based nanocarriers have been increasingly explored [15]. Lipid nanodispersions enable the encapsulation of lipophilic molecules, improve their stability, and may enhance their interaction with biological interfaces. Additionally, such systems may improve interaction with the skin barrier and support controlled release of active compounds [18].

The integration of hydrogel matrices with lipid nanodispersions results in multicomponent hybrid systems that can act as versatile carrier platforms for the simultaneous incorporation of hydrophilic and lipophilic bioactive compounds [15]. Such systems may exhibit synergistic properties arising from the combination of structural features of hydrogels and the carrier functionality of lipid nanostructures, including controlled release and prolonged interaction with the application surface [19,20,21,22,23,24,25,26,27,28].

According to current literature, hybrid systems combining nanocarriers with hydrogel matrices represent advanced platforms for controlled and sustained dermal delivery of active compounds. Such systems enable modulation of release kinetics, thereby prolonging the residence time of active substances within the skin and enhancing their therapeutic efficacy. The controlled release behavior arises from the structural properties of both components: nanocarriers act as reservoirs for lipophilic compounds, while the hydrogel network governs their diffusion and release into the surrounding environment [29].

In the initial stage of application, nanoparticles containing lipophilic active ingredients, typically localized within the internal phase of nanoemulsions or lipid nanocarriers, are progressively released into the hydrogel matrix. Subsequently, the cross-linked polymeric structure of the hydrogel acts as a secondary diffusion barrier. This three-dimensional network is capable of entrapping dispersed nanoparticles and limiting their mobility, resulting in a sustained and controlled release profile. The rate of release is strongly dependent on the crosslinking density, porosity, and swelling behavior of the hydrogel, which collectively regulate the diffusion of both nanoparticles and the encapsulated active substances [30].

Importantly, this dual-stage release mechanism significantly prolongs the exposure of the skin to lipophilic bioactives, enhancing their penetration and retention within the stratum corneum. Nanocarriers further contribute to improved dermal delivery by facilitating closer interaction with the skin surface and increasing the local concentration gradient of the active compound, which promotes diffusion into deeper layers of the epidermis [29].

In addition to controlled delivery, nanocarrier–hydrogel systems exhibit pronounced effects on skin barrier function. The hydrogel matrix forms a continuous, hydrophilic film on the skin surface, which enhances water retention and reduces transepidermal water loss (TEWL). Simultaneously, lipid-based nanoparticles can create a semi-occlusive layer that further limits water evaporation and improves skin hydration. This occlusive effect has been shown to significantly increase stratum corneum hydration and elasticity, while supporting barrier regeneration processes [31]. The prolonged residence time of the formulation on the skin, facilitated by the rheological properties of hydrogels, additionally enhances bioadhesion and ensures sustained contact between the active compounds and the skin surface.

In this context, we developed a multicomponent hybrid system based on an alginate hydrogel matrix combined with a lipid nanodispersion containing selected bioactive compounds, including silymarin, bakuchiol, and spirulina. The novelty of the proposed system lies in the integration of hydrogel and lipid nanocarrier phases into a single platform enabling the incorporation of compounds with different physicochemical properties and the combination of physical and functional effects.

The first stage of the research, focusing on the design, preparation, and physicochemical characterization of the developed systems (including structural, mechanical, thermal, and cytotoxic properties), has been reported in our previous work [32]. In contrast, the present study is dedicated to the evaluation of the safety and functional performance of the selected formulation.

The aim of this study was to assess the biological and functional properties of the developed hybrid system, including its effects on skin hydration, transepidermal water loss (TEWL), erythema, elasticity, and sensitivity, as well as selected biochemical parameters such as inhibition of collagen and elastin glycation and stimulation of type I procollagen synthesis. Although such systems may be considered for supportive skincare applications, including post-procedural conditions, the present study focuses on their material properties and functional performance. Further studies involving compromised or post-treatment skin are required to confirm their effectiveness in such conditions.

## 2. Materials and Methods

### 2.1. Materials

To obtain the multibiocomponent hybrid hydrogels, a variety of specific materials were required. Medium-viscosity sodium alginate (SA) derived from brown algae and silymarin was procured from Merck (Darmstadt, Germany). Avantor Performance Materials (Gliwice, Poland S.A.) provided the anhydrous calcium chloride.

Several botanical and cosmetic ingredients, namely spirulina and bakuchiol, were purchased from Zrób Sobie Krem (Prochowice, Poland), a domestic vendor of herbal raw materials. Similarly, ECOSPA (Warszawa, Poland) supplied the lavender hydrolate along with Olivem 1000, an emulsifying blend of cetearyl olivate and sorbitan olivate. Finally, borage oil was sourced from Provital (Luboń, Poland).

### 2.2. Methods of Preparation

**Figure 1:**
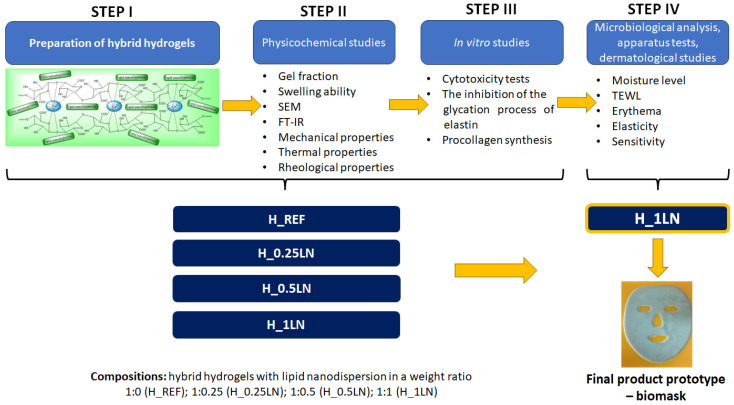
General research procedure—preparation, methodology, and results of STEPS I-II were presented in previous studies [32].

All the details regarding the preparation method of the lipid nanodispersion (LN; Component A), polymeric matrix (SA(S); Component B), and hybrid hydrogels, as well as physicochemical and in vitro studies, were described in the previous article (STEPS I-II, Figure 1) [32]. As the final product, multibiocomponent hybrid hydrogels with lipid nanodispersion in a weight ratio 1:0 (H_REF), 1:0.25 (H_0.25LN), 1:0.5 (H_0.5LN), 1:1 (H_1LN) were obtained [33]. The general procedure for conducting research is presented in Figure 1.

#### 2.2.1. Preparation of the Lipid Nanodispersion (LN; Component A)

The nanoemulsion systems employed in this study were composed of borage oil, bakuchiol, silymarin, deionized water, and Olivem 1000 as the emulsifying agent. Formulations were prepared using a high-energy emulsification method based on ultrasonication. Initially, a coarse pre-emulsion was obtained by combining the aqueous phase with the oil phase—consisting of borage oil, bakuchiol, silymarin, and the emulsifier—under controlled thermal conditions (T ≤ 70 °C). The mixture was homogenized using magnetic stirring (IKA C-MAG HS 7, IKA Poland Sp. z o.o., Warszawa, Poland) at 500 rpm for 5 min to ensure adequate dispersion of the components. Subsequently, the pre-emulsion was subjected to high-energy processing using a probe-type ultrasonic homogenizer UP200 Ht (Hielscher Ultrasonics GmbH, Teltow, Germany) in order to reduce droplet size and obtain a stable lipid nanodispersion [32,33].

#### 2.2.2. Preparation of Polymeric Matrix (SA(S); Component B)

A sodium alginate solution, derived from brown algae, was prepared by dissolving the polymer in lavender hydrolate at an elevated temperature of 70–75 °C. Following complete dissolution, spirulina was incorporated into the polymeric system. The resulting mixture was then subjected to intensive homogenization using a mechanical stirrer at a constant v = 1500 rpm for 3 min, while maintaining the temperature within the range of 20–25 °C over 3 min [32,33].

#### 2.2.3. Hybrid Hydrogels Preparation

Component B was combined with Component A at weight ratios of 1:1, 1:0.5, and 1:0.25 under mechanical stirring (1500 rpm, 2 min, 22–25 °C). The mixtures were then transferred to Petri dishes and cross-linked using a 5% (*w*/*w*) calcium chloride solution (Component C) at a 2:1 weight ratio relative to the formulation. The resulting hydrogels were rinsed with distilled water to remove excess cross-linking agent. Final multicomponent hybrid hydrogels containing lipid nanodispersion were obtained at ratios of 1:0 (H_REF), 1:0.25 (H_0.25LN), 1:0.5 (H_0.5LN), and 1:1 (H_1LN) [32,33].

### 2.3. In Vitro Evaluation

#### 2.3.1. Collagen Glycation Assay

The glycation process of this protein was measured with a commercial kit: the collagen glycation test (Cosmo Bio Co., Ltd., Tokyo, Japan), following the manufacturer’s instructions. Before the experiments, the black 96-well plate was coated with collagen solution and incubated at 37 °C and high humidity for 24 h. Afterward, the appropriately prepared bio-hydrogel masks (cropped disks approximately 6.5 mm in diameter) were inserted with the assay buffer into the wells. Additionally, the freshly prepared solutions of aminoguanidine (positive standard) at concentrations of 0.8 mM, 4 mM, and 20 mM were added to other wells. Then, the solution of glyceraldehyde (500 mM) was added to each well to induce the glycation process. The fluorescence intensity was immediately measured with a multi-plate reader VarioSkan LUX (Thermo Fisher, Waltham, MA, USA) at an excitation wavelength of 370 nm and an emission wavelength of 440 nm. Then, the plate was incubated at 37 °C and high humidity for 6 days. In the meantime, the fluorescence intensity was measured every two days. T The experiments were performed in quadruplicate.

#### 2.3.2. Elastin Glycation Assay

Elastin glycation was quantified using a commercial elastin glycation assay kit (Cosmo Bio Co., Ltd., Tokyo, Japan), according to the manufacturer’s protocol. The elastin solutions were added to each well of the black 96-well plate. The appropriately prepared bio-hydrogel masks (cropped disks approximately 6.5 mm in diameter) were immediately inserted with the assay buffer into the wells. Additionally, the freshly prepared solutions of aminoguanidine (positive standard) at concentrations of 0.8 mM, 4 mM, and 20 mM were added to other wells. Then, the solution of glyceraldehyde (500 mM) was added to each well to induce the glycation process. The fluorescence intensity was immediately measured with a multi-plate reader VarioSkan LUX (Thermo Fisher Scientific, Waltham, MA, USA) at an excitation wavelength of 370 nm and an emission wavelength of 440 nm. Then, the plate was incubated at 37 °C and high humidity for six days. In the meantime, the fluorescence intensity was measured every two days. The experiments were performed in quadruplicate.

#### 2.3.3. Procollagen Type I C-Peptide (PIP) Synthesis

The NHDF cell line was purchased from PromoCell. Normal fibroblasts were cultured in fibroblast growth medium with a low-serum content (PromoCell, PromoCell GmbH, Heidelberg, Germany) containing a mix of antibiotics-penicillin and streptomycin (1% *v*/*v*; Gibco, Waltham, MA, USA). Prior to the start of the experiments, NHDF cells were seeded into 24-well plates at 35,000 cells per well and incubated at 37 °C for 48 h. Then, bio-hydrogel masks were prepared by cutting disks approximately 1 cm in diameter and inserted into each well containing the medium. The tested materials with cells were incubated at 37 °C for 1 h. Afterwards, the tested masks were removed and the cells were trypsinized and neutralized with the culture medium. To extract the intracellular contents, fibroblast cells were placed in Eppendorf tubes, centrifuged, and lysed on ice. The lysis medium consisted of a complete RIPA buffer enriched with 0.5 M EDTA, alongside Halt protease and phosphatase inhibitor cocktails sourced from Thermo Scientific. Following this step, total protein concentrations were established using a standard BCA Protein Assay Kit (Thermo Scientific, Waltham, MA, USA) exactly as instructed by the manufacturer. For the quantification of procollagen generation, equal 4.5 µg protein fractions from every sample were analyzed utilizing Takara Bio’s Procollagen Type I C-Peptide (PIP) enzyme immunoassay kit. The analytical procedure began by dispensing the samples, reference standards, and an antibody-POD conjugate directly into a microplate already treated with anti-PIP monoclonal antibodies. This prepared plate was then incubated for three hours at 37 °C. Once the initial incubation finished, the wells were washed four times to remove unbound materials. A TMB (3,3′,5,5′-tetramethylbenzidine) substrate was then introduced, prompting a 15 min reaction at room temperature. Finally, a stop reagent was added to halt the process, and the absorbance values were instantly captured at 450 nm with a VarioSkan LUX (Thermo Fisher Scientific, Waltham, MA, USA) microplate reader. The exact concentrations of the synthesized PIP were computed by referencing the plotted standard curve. The experiments were performed in quadruplicate.

### 2.4. Microbiological Quality Assessment

Article 3 of Regulation (EC) No. 1223/2009 states that cosmetic products must be safe for human health under normal or reasonably foreseeable condition of use. As a result, a cosmetic product must be microbiologically pure under specific microbiological criteria, which depend on the type of the product and its intended use.

In terms of microbiological purity, the bio-mask was tested following the standard PN-EN ISO 17516: 2014-11 Cosmetics–Microbiology–Microbiological limits standard and it belongs to category II of cosmetics [34]. This means that the maximum acceptance limit for the general number of aerobic mesophilic microorganisms is 2.0 × 10^3^ CFU/g, with specific microorganisms (*Pseudomonas aeruginosa*, *Staphylococcus aureus*, *Candida albicans*, *Escherichia coli*) absent in 1 g.

To ensure the quality of the developed bio-mask and the safety of the user, microbiological purity tests and a challenge test were carried out. The tests were conducted in an accredited cosmetics laboratory, Cosmetic Analysis Laboratory NOVA (Podłęże, Poland).

The standardized and reproducible test methods were used to assess the microbiological purity: PN-EN ISO 16212:2017-08 Cosmetics–Microbiology–Enumeration of yeast and mold [35], PN-EN ISO 21149:2017-07 Cosmetics–Microbiology–Enumeration and detection of aerobic mesophilic bacteria [36], PN-EN ISO 18416:2016-01 Cosmetics–Microbiology–Detection of *Candida albicans* [37], PN-EN ISO 21150:2016-01 Cosmetics–Microbiology–Detection of *Escherichia coli* [38], PN-EN ISO 22717:2016-01 Cosmetics–Microbiology–Detection of *Pseudomonas aeruginosa* [39], PN-EN ISO 22718:2016-01 Cosmetics–Microbiology–Detection of *Staphylococcus aureus* [40].

The challenge tests were carried out when the cosmetic product was being developed during the laboratory stage to confirm the preservative efficacy. The challenge test of a cosmetic product is a simulation of secondary infection that may occur when the ready product is used by the consumer. The bio-mask challenge test was performed under the European Standard PN-EN ISO 11930:2019-03 Cosmetics–Microbiology–Evaluation of the antimicrobial protection of a cosmetic product [41], by using the following reference strains purchased from ATCC: *Pseudomonas aeruginosa* ATCC 9027, *Staphylococcus aureus* ATCC 6538, *Escherichia coli* ATCC 8739, *Candida albicans* ATCC 10231, *Aspergillus brasiliensis* ATCC 16404. It confirmed that the tested product was microbiologically pure. For each tested microorganism, the cosmetic formulation was challenged with a standardized inoculum, and microbial counts were determined at defined time points under controlled temperature conditions. Incubation conditions: 32.5 °C ± 2.5/72 h (bacteria, *Candida albicans*); 22.5 °C ± 2.5/5 days (*Aspergillus brasiliensis*). The seeding date for bacteria and yeasts is the 7th day, 14th day, and 28th day; for filamentous fungi it is the 14th day and 28th day. The following substrates were used in the study: TSA (Tryptic Soy Agar), SDA (Sabouraud Dextrose Agar Medium), PDA (Potato Dextrose Agar), and a neutralizer: Eugon broth with Lecithin, Polysorbate 80, and Triton X-100.

### 2.5. Instrumental Tests

The study was performed in accordance with Regulation (EC) No 1223/2009, Cosmetics Europe–The Personal Care Association “Guidelines for the Evaluation of the Efficacy of Cosmetic Products 2008”, World Medical Association (WMA) Declaration of Helsinki–Ethical Principles for Medical Research Involving Human Subjects and Polish Cosmetic Law 4 October 2018, Journal of Laws of 2018, no. 2227.

The test was carried out in the laboratory of SkinLab P.S.A. (Cracow, Poland) under an internal study procedure for conducting tests (PO-08, Conducting Tests), and technical instructions (I06/PO-08, Instrumental Tests). The subjects participating in the tests were selected following the technical instructions (I01/PO-08, Qualification of Subject Test Subjects to the Study). All of the selected subjects met the requirements for inclusion in the study and signed a consent of voluntary participation in the study. They were also informed about the aim of the study, i.e., an objective assessment of the effect of the product on the condition of the skin as well as an assessment of the declared functional properties of the preparation, the method used in the study, and possible adverse effects of the product. The subjects were under the constant care of a dermatologist during the study.

The study group consisted of five women, aged 23–35, with normal skin type. The study was conducted at home. For this purpose, the subjects received the tested product, and they were informed about how to use it.

According to the European Parliament and of the Council, Cosmetics Europe–The Personal Care Association “Guidelines for the Evaluation of the Efficacy of Cosmetic Products 2008” instrumental tests are performed with instruments that can precisely measure given parameters, according to a predefined protocol, following product application in human participants. Skin parameters were measured with Derma Lab Combo and ASW 300 measuring devices (BECHTOLD & CO SP. Z O.O., Łódź, Poland). The device allows the analysis of the skin condition with sensors that measure: hydration, TEWL, erythema, elasticity, and sensitivity. The average value of a tested parameter was recorded from the measurements in all human subjects. The final result includes the percentage of change in the parameter value concerning the baseline value. A positive result for TEWL, erythema, and skin sensitivity is a decrease in value, while an increase in the value is a positive result for hydration and elasticity.

### 2.6. Statistical Analysis

All quantitative data are presented as the mean ± standard deviation (SD) from at least three independent experiments. Statistical analysis was performed using GraphPad Prism software version 10 (GraphPad Software, San Diego, CA, USA). For the collagen and elastin glycation assays, a two-way analysis of variance (two-way ANOVA) followed by Dunnett’s post hoc test was applied to evaluate differences between the tested bio-hydrogel masks and the control (untreated protein) at individual time points. Differences were considered statistically significant at *p* < 0.05. Statistical significance levels are indicated in the figures as follows: * *p* < 0.05; ** *p* < 0.01; **** *p* < 0.0001.

## 3. Results

### 3.1. Results of the Microbiological Study and the Challenge Test

The results of the microbiological purity tests of the bio-mask carried out following PN-EN ISO 17516:2014 [34] are shown in Table 1. The obtained results indicate that the tested sample met the requirements of category II cosmetics. The analyzed sample does not contain pathogenic strains, such as *Pseudomonas aeruginosa*, *Staphylococcus aureus*, *Escherichia coli*, or *Candida albicans*. The total number of mesophilic aerobic microorganisms was 5.3 × 10^2^ CFU/g or mL and does not exceed the permissible value of 1 × 10^3^ CFU/g or mL.

The effectiveness of antimicrobial protection in cosmetic products can be assessed with a challenge test. The choice of the method for this test depends on the type of cosmetic product, its ingredients, and the production process. In this study, the challenge test was carried out under the PN-EN ISO 11930:2019-03 standard [41]. In this method, each test strain is tested separately. The formulation samples are contaminated once and left for a specified duration. This way, the response speed of the preservative system is checked. There are two acceptance criteria: A and B. If the cosmetic product meets the requirements of the A profile, this means that the used preservative system effectively protects the formulation against the growth of microorganisms that can pose a risk to users, and no additional protective factors are required. When the cosmetic product meets the requirements of the B profile, the protection level for the formulation is acceptable if the risk analysis shows the presence of factors that are unrelated to the cosmetic substance and which ensure the acceptable microbiological risk of the cosmetic product, e.g., packaging with a dispenser that limits the contact of the formulation with air and the skin microbiota. The result is expressed as the calculated reduction rate of the number of microorganisms after a given time since contamination. During the study, the effectiveness of the neutralizer is also determined. It neutralizes the effect of the preservative used in the formulation and does not inhibit the growth of the tested bacteria and fungi [42,43]. The acceptance criterion for the neutralization level of preservatives is shown in Table 2.

The reduction values (Table 3) expressed in logarithmic units were compared with minimum values necessary to meet one of the two criteria (A or B) and, as a result, it was concluded that criterion A was met. The obtained bio-mask with the preservation system, INCI name: Aqua (and) Glycerin (and) Sodium Levulinate (and) Sodium Anisate; Sodium Benzoate, is protected against microorganism growth, so it does not pose a risk to its users.

### 3.2. In Vitro Studies

#### 3.2.1. Antiglycation Potential

**Figure 2:**
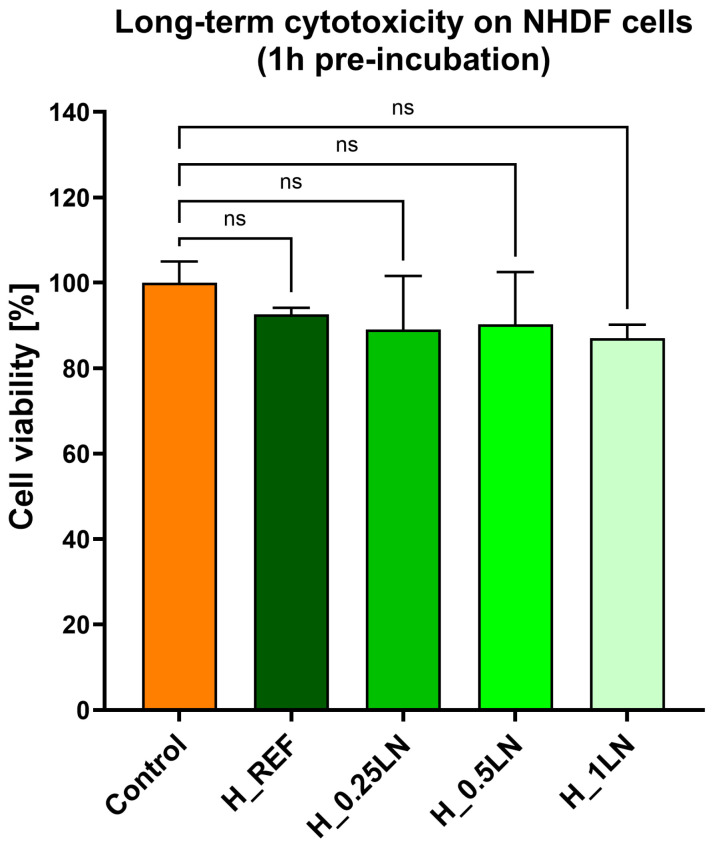
Long-term cytotoxicity on fibroblast cells (NHDF cell line) after 24 h. The cells were pre-incubated with tested bio-hydrogel masks for 1 h. Data expressed as mean ± SD (*n* = 3). The statistical analysis was performed using a two-way ANOVA with Dunnet’s post hoc test; ns—non-significant compared to the control (untreated cells).

To evaluate the functional efficacy of the formulated bio-masks, their ability to inhibit the glycation of key structural dermal proteins was investigated. However, prior to assessing this antiglycation potential, the safety profile of the samples had to be established. As detailed in our earlier work [32], tests were conducted on human fibroblast-type cells to determine the toxicity profile of the tested samples. As shown in Figure 2, none of the tested bio-masks affected the viability of the fibroblast cells.

**Figure 3:**
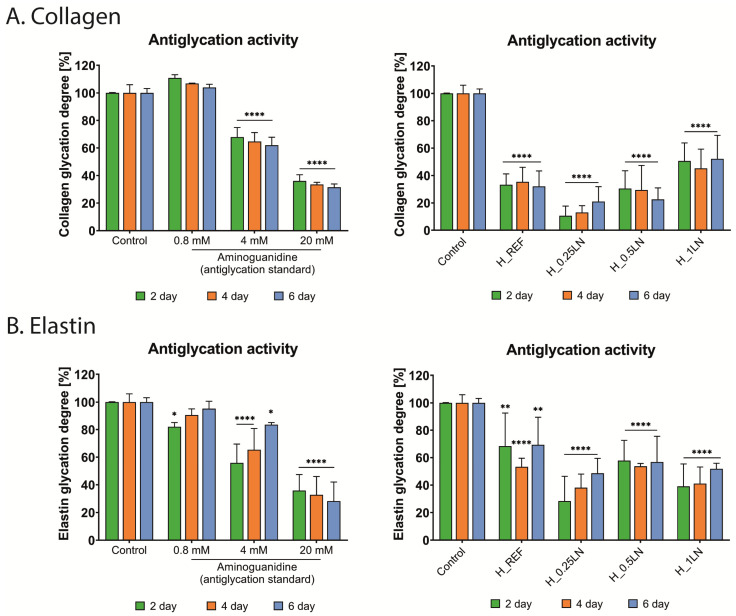
The influence of the tested bio-hydrogel masks and aminoguanidine on the glycation process of collagen (**A**) and elastin (**B**). Data expressed as mean ± SD (*n* = 4). The statistical analysis was performed using a two-way ANOVA with Dunnet’s post hoc test: * *p* < 0.05, ** *p* < 0.01, **** *p* < 0.0001, compared to the control (untreated protein).

The antiglycation potential of bio-hydrogel masks against collagen and elastin was estimated using fluorescent assays. The formation of non-cross-linked advanced glycation end products (AGEs) in the above-mentioned proteins was induced by glyceraldehyde addition. The assays were conducted for six days, with the fluorescence intensity of AGEs production monitored simultaneously. The results are presented in Figure 3.

The reference in both tests was aminoguanidine at doses 0.8, 4.0, and 20.0 mM, which exhibited antiglycation potential. Namely, the glycation process of collagen was effectively inhibited by aminoguanidine at the two highest concentrations (Figure 3A). Nearly 60% inhibition of collagen glycation was registered after two days of incubation with a reference at a concentration of 20.0 mM. This trend was maintained for a further four days of incubation. Interestingly, a similar scale of glycation collagen inhibition was observed for H_REF and H_0.5LN masks after two days. In the case of H_REF, the low level of collagen glycation degree was sustained for six days, while the antiglycation effect of the bio-hydrogel mask, which contained a ratio of 1:0.5 of the polymeric matrix to lipid nanodispersion, was aggravated. After six days of incubation with H_0.5LN, the inhibition of collagen glycation increased to nearly 80%. However, the strongest effect was observed for H_0.25LN, where the inhibition was almost 90% after two days of incubation. On the other hand, the inhibitory effect weakened in the following days. The explanation of this phenomenon may be related to the degradation of the H_0.25LN bio-hydrogel mask. Nevertheless, the beneficial effect of the tested bio-hydrogel masks on collagen, which counteracts the weakening of its structure, is undeniable. Such excellent antiglycation effects obtained for the above-mentioned samples overshadow the last sample H_1LN, for which the degree of inhibition was about 50% and did not significantly differ during the incubation time. However, these results, too, are promising and qualify the tested masks for further research.

In the case of the second analyzed protein, elastin, the inhibitory effect on the glycation process by bio-hydrogel masks was not as remarkable (Figure 3B). In turn, the aminoguanidine induced an inhibitory effect at a concentration of 20.0 mM that was rather similar to that for collagen. Namely, a reduction in the degree of elastin glycation after two days of about 60% was observed. Over the following four days, the inhibition of elastin glycation increased to about 70%. In the case of the tested bio-hydrogel masks, the strongest inhibitory effect was registered also for H_0.25LN after two days (about 70% of inhibition). In addition, a reduction in the degree of elastin glycation of above 60% was noted for the H_1LN bio-hydrogel mask after two and four days of incubation. A slightly lower result was obtained for the bio-hydrogel mask with a formulation 1:0.5 of the polymeric matrix to lipid nanodispersion–H_0.5LN (below 50% of inhibition). For this formulation of the mask, the inhibitory effect against elastin glycation was sustained in the following four days of incubation. In the case of the remaining masks, the effect was weakened. Despite this, the obtained results for the bio-hydrogel masks with different ratios of the polymeric matrix to lipid nanodispersion were better than for the reference mask–H_REF. These findings may indicate beneficial effects of the addition of lipid nanodispersion in the context of maintaining the structural, elastic, and functional integrity of the skin.

#### 3.2.2. Procollagen Synthesis

**Figure 4:**
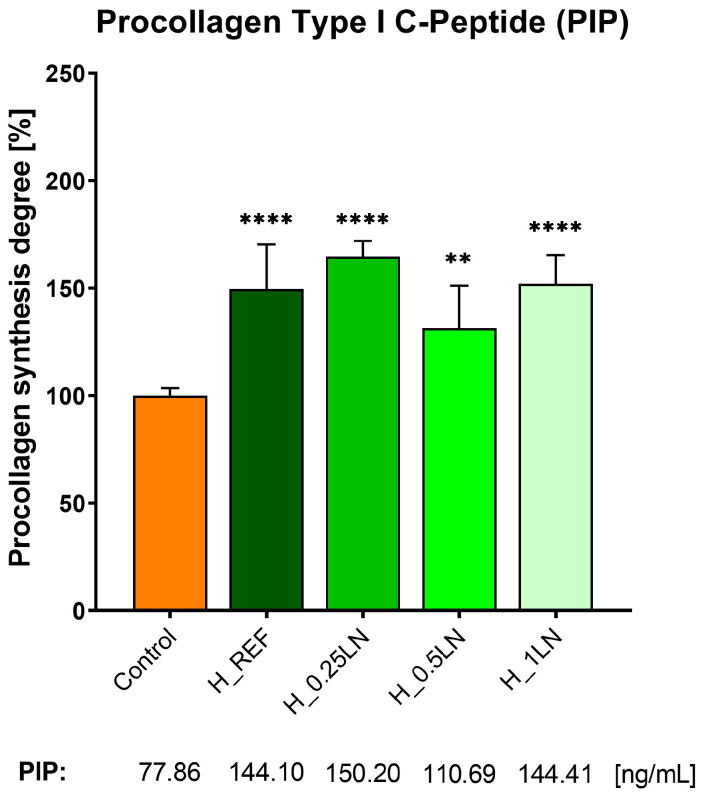
Procollagen Type I C-Peptide (PIP) synthesis in fibroblast cells, which were incubated with tested bio-hydrogel masks for 1 h. Data expressed as mean ± SD (*n* = 4). The statistical analysis was performed using a one-way ANOVA with Dunnet’s post hoc test: ** *p* < 0.01, **** *p* < 0.0001, compared to the control.

In the next step of research, the influence of the bio-hydrogel masks on procollagen synthesis was evaluated. For this purpose, an immunoenzymatic assay was used, in which the production of the procollagen type I C-peptide (PIP) was quantified with the antibodies in fibroblast cells. It is worth mentioning at this point that the collagens building connective tissue, organs, skin, hair, nails, and well as blood vessels are formed from precursor compounds, which are procollagens [44]. These compounds contain additional peptide sequences that facilitate the formation of the final conformational structure of the collagen triple helix in the endoplasmic reticulum. Once the final collagen structure is obtained, the peptide sequences are released into the free form [45]. The content of the free forms of these peptides is directly proportional to the amount of collagen synthesized [46]. Many studies show that serum concentration PIP reflects the mechanism of fibrogenesis, which leads to wound healing and repair in normal conditions [47,48]. As depicted in Figure 4., our ELISA assays revealed that procollagen synthesis was stimulated when fibroblast cells were incubated with the tested bio-hydrogel masks. The degree of procollagen synthesis was more than 150% compared to the control (cells without bio-hydrogel mask). One exception was a bio-hydrogel mask with a formulation 1:0.5 of the polymeric matrix to lipid nanodispersion–marked as H_0.5LN. Due to the cosmetic application, the tested bio-hydrogels were incubated for one hour with NHDF cells. It is worth noting that the masks containing lipid nanodispersion: H_0.25LN and H_1LN increased the procollagen synthesis to a greater extent than the H_REF mask. The calculated PIP concentration in NHDF cells was 150.2 and 144.41 ng/mL for H_0.25LN and H_1LN, respectively.

### 3.3. Results of Instrumental Tests

**Figure 5:**
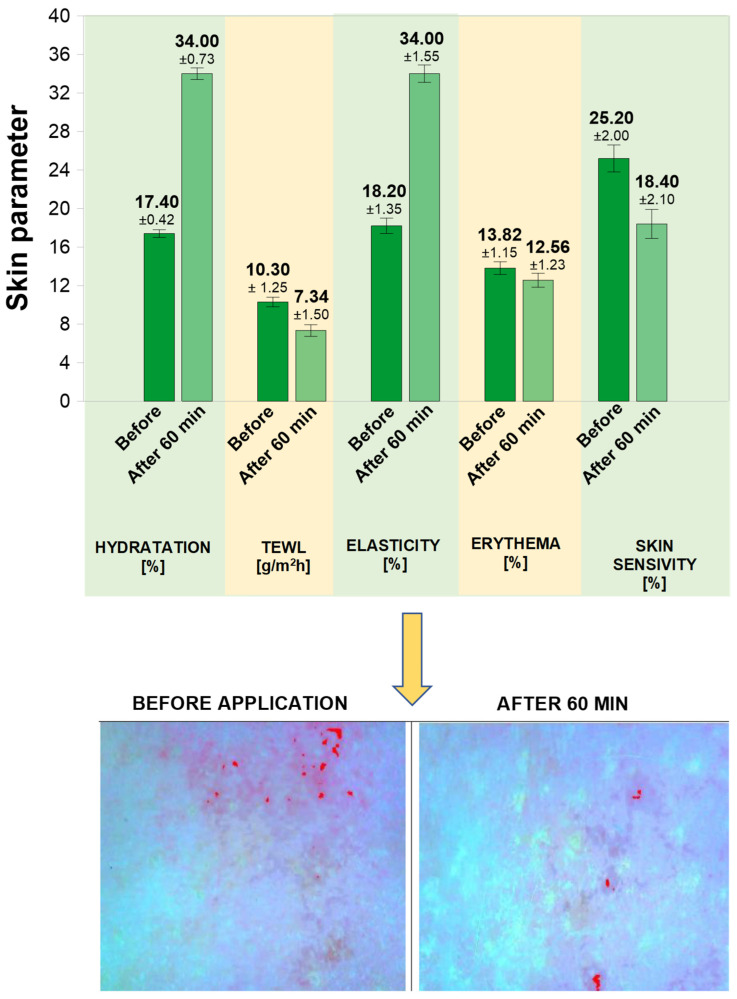
The mean skin parameter value obtained from the measurements carried out in all subjects: hydration, TEWL, elasticity, erythema, and skin sensitivity.

During the study, the following skin parameters were assessed: hydration, TEWL, erythema, elasticity, and sensitivity. The results are shown in Figure 5.

The assessment of selected skin parameters before application of the bio-mask and 60 min thereafter allowed for a preliminary evaluation of its effects on the skin and its declared functional properties. The observed increase in skin hydration after 60 min from bio-mask application by +91.01% (from 17.40 to 34.0) suggests that the product has a moisturizing effect. The improved skin hydration is also shown by decreased TEWL by 28.74% (from 10.3 to 7.34). After 60 min from the application of the bio-mask, the elasticity increased from 18.20 to 34.0, i.e., by +86.81%.

The erythema value informs about the degree of skin redness. The higher the value of this parameter, the more the skin is reddened. The observed decrease in the erythema value by 9.12% compared to the initial value (from 13.82 to 12.56) as well as the decrease in the value of skin sensitivity from 25.20 to 18.40, i.e., by −26,98%, may indicate a potential soothing effect of the product.

In summary, the results suggest a trend toward improved skin parameters as early as 60 min after application. Nevertheless, given the exploratory nature of the study, the small number of participants, and the lack of controlled conditions, these observations should be considered preliminary. The effects may be related to the combined action of hydrophobic active substances incorporated as lipid nanoparticles within a polymer matrix and the hydrophilic structure of the hydrogel. The hydrogel component may contribute to skin hydration and barrier function by forming a hydrophilic film that enhances water retention and reduces transepidermal water loss (TEWL), while the occlusive properties of lipid nanoparticles may further support this effect. This potential synergistic mechanism—combining occlusion, humectancy, and controlled release—could create a microenvironment conducive to skin regeneration and repair [49], although further well-controlled studies on a larger group of probants are required to confirm these findings.

## 4. Discussion

The investigated bio-mask represents a prototype formulation; therefore, the study was designed as proof-of-concept investigation, with particular emphasis on safety assessment in accordance with Regulation (EC) No 1223/2009, rather than on full clinical validation.

The developed bio-mask prototype is a hybrid combination of lipid nanodispersion with hydrogel, which in its formula contains a concentrate of nourishing, moisturizing, and stimulating skin regeneration substances, including silymarin, bakuchiol, and spirulina.

Those natural bioactive components play a very important role in skin regeneration due to their anti-inflammatory and antioxidant properties, especially since excessive production of reactive oxygen species (ROS) due to inflammation is one of the obstacles to skin regeneration [50].

Multiple investigations demonstrate that silymarin shields the skin from damage caused by chemicals and ultraviolet B radiation. This defense mechanism, observed in both animal and cellular models, relies on its strong antioxidant and anti-inflammatory properties [51]. Research by Toklu and colleagues [52] highlighted how giving silymarin either topically or systemically helps lessen oxidative damage following burn trauma. Skin wounds naturally spark an inflammatory reaction, which overstimulates neutrophils and leads to oxidative harm in the surrounding cells. Burn injuries in particular are driven by reactive oxygen metabolites (ROM) alongside active macrophages and neutrophils. This triggers lipid peroxidation, a destructive cycle that tears down cellular membranes. Fortunately, antioxidants like silymarin act as a barrier against this type of trauma-induced tissue stress [52]. Moreover, Tabari’s team [53] discovered that applying a silymarin ointment sped up the recovery of wounds, visibly reducing redness, discharge, and swelling. During the later stages of tissue repair, higher doses of the extract effectively dropped the levels of macrophages and lymphocytes while pushing up fibrocyte numbers [53]. Sharifi and associates [54] also looked into how silymarin (*Silybum marianum*) interacts with human skin fibroblasts using a lab-based wound model. Their findings indicated that treating the cells with the extract beforehand offered significant protection against hydrogen peroxide-induced harm. Lastly, Ashkani-Esfahani et al. [51] noted that treating wounds with a silymarin gel accelerates recovery by boosting collagen creation, fibroblast multiplication, and general tissue regeneration. The compound was even shown to enhance new blood vessel formation around the healing site.

In the case of bakuchiol, Bluemke et al. [55] observed that a monoterpene phenol displayed a significant increase in epidermis regeneration. Multidirectional effects of bakuchiol such as antioxidative, anti-inflammatory, increased cell activity, the expression of critical extracellular matrix (ECM) components, and improved epidermal regeneration were investigated. Obtained studies demonstrated that bakuchiol decreases oxidative stress, prevents mitochondrial lipid peroxidation, and protects mitochondrial function. Bluemke and co-workers’ findings indicated that bakuchiol might support skin regeneration and repair processes by directly upregulating keratinocytes and indirectly increasing fibroblast proliferation [55].

Regarding blue-green algae, Jung and co-workers [50] demonstrated that spirulina extracts help regulate both internal and external reactive oxygen species (ROS). The extract achieves this by strengthening the natural antioxidant defenses of fibroblasts when they face oxidative stress. By keeping ROS levels in check at the injury site, spirulina minimizes inflammation and accelerates tissue repair. Animal studies further validated these findings, showing that polycaprolactone (PCL) nanofibers infused with the extract serve as highly effective regenerative wound dressings [50].

In a 2015 study, Delsin’s group [56], explored the topical benefits of this microalga. They found that applying it as a gel-cream upgrades the epidermal architecture and deeply hydrates the skin, strengthening its natural barrier. Subjects experienced noticeably higher moisture levels in the stratum corneum alongside a drop in both transepidermal water loss (TEWL) and surface sebum. The formulation also improved dermis density and the proper spread of keratinocytes [56]. Thanks to its rich peptide profile, spirulina actively encourages the body to produce more collagen and glycosaminoglycans while prompting fibroblasts to multiply [57].

Despite their therapeutic or cosmetic applications, active molecules face some challenges, particularly their limited ability to penetrate through the SC, thus compromising their permeation. To overcome this problem is very important to develop an optimal carrier [58].

Bio-mask as a carrier is composed of a hybrid combination of lipid nanodispersion, containing lipophilic bioactive substances (silymarin and bakuchiol) with hydrogel containing hydrophilic bioactive (spirulina). The in vitro studies confirmed that the addition of the silymarin/bakuchiol-incorporated lipid dispersion to the spirulina-incorporated hydrogel matrix strongly enhanced the inhibition of collagen and elastin glycation in comparison to the reference sample (hydrogel matrix). Reduction in the degree of elastin glycation of above 60% and 50% inhibition of collagen glycation was noted for the final formulation (H_1LN) after two and four days of incubation. Moreover, the bio-mask containing lipid nanodispersion increased the procollagen synthesis to a greater extent compared to basic hydrogel and more than 150% compared to the control (cells without bio-hydrogel mask). These highly promising results highlight the formulation’s anti-aging potential. While collagen and elastin are essential for skin elasticity and resilience [59], they are highly susceptible to degradation through glycation, a process driven by non-enzymatic Maillard reactions [60,61]. By effectively preventing the accumulation of these harmful byproducts and the subsequent stiffening of the extracellular matrix [62], the addition of lipid nanodispersion significantly contributes to maintaining the structural, elastic, and functional integrity of the skin.

As the literature reports [63] the epidermal barrier is disrupted, red, and swollen after laser treatment, and, in particular, the TEWL is increased and hydration is decreased, which triggers inflammation. Therefore, topical formulations used after laser therapy should provide an increase in hydration and elasticity, restore surface skin lipids, and decrease inflammation alleviating symptoms of dry skin during treatment. These preparations should also have a soothing effect.

Preliminary instrumental analysis suggested that the formulation increased skin hydration by approx. 90%, reduced TEWL by approx. 28%, reduced erythema by approx. 9%, increased skin elasticity by approx. 89%, and decreased skin sensitivity by approx. 27%. The reduced transepidermal water loss may suggest that skin barrier integrity has improved and it may be symptomatic of an occlusive effect of the formulation in use, which is a desirable effect for sheet masks. Properly moisturized skin also becomes more elastic. Added value is the form of a ready-made sheet that allows for single, hygienic use and ensures ease of application—there is no need to prepare the product and wash the skin after its application [7,13]. According to the research by Perugini et al. [64], the erythema level also can be used to assess the mask tolerability, while the TEWL parameter may be applied to assess the irritant effect of the facial mask. The observed decrease in erythema with the simultaneous decrease in TEWL may suggest that the obtained bio-mask does not cause irritation or redness when in contact with the skin, which is consistent with the results from the dermatological tests.

Taking into consideration the above-mentioned information, the mechanism of bio-mask action may be based on two phenomena. The mask in the form of a hybrid hydrogel creates a hydrophilic barrier between the skin and the environment, preventing transepidermal water loss. The hydrogel matrix may also contribute to a soothing and cooling effect, helping to reduce skin irritation and erythema [14].

The incorporation of lipid nanodispersion may support the interaction of lipophilic bioactive compounds with the skin surface and potentially enhance their availability; which may result in a visible improvement in the skin’s hydration, elasticity, sensitivity, and hydration levels [23,65]. Future studies should include dedicated permeation experiments to validate this mechanism, such as Franz diffusion cell assays, tape-stripping analysis, confocal microscopy imaging, and investigations using reconstructed human epidermis or excised skin models.

However, it must be emphasized that these instrumental in vivo tests were conducted on the relatively small number of participants, which may limit the generalizability of these findings, as well as the strength of the conclusions. Additionally, the study group consisted exclusively of young women with healthy skin. Such a population does not reflect the target cohort-post laser patients-who typically exhibit compromised barrier function, heterogeneous skin types, broader age ranges, and mixed sex distribution.

Another limitation of the in vivo study is the lack of a control group, including a reference formulation without the addition of lipid nanodispersion (reference mask), as well as absence of a split-face design. This limits the ability to attribute the observed effects solely to the tested formulation, as the occlusive effect typically associated with application of sheet masks must be considered as a contributing factor. However, it should be noted that the obtained results for antiglycation potential and procollagen synthesis were more favorable compared to the reference mask.

Regarding the study protocol, a single application of the product was carried out at home, the application time was 60 min. This was consistent with the application time of products used after laser therapy, where the conventional cooling mask is applied to the face, neck, or neckline immediately after the procedure for a period of 20 to 60 min. Although a split-face design was not applied, the chosen study design (single application on the skin, performed by participants at home) allowed for full-face application, thereby more closely reflecting real-life usage conditions. Nevertheless, it should be noted that a single application does not allow for the assessment of long-term effects or repeatability of the observed outcomes.

Considering the above limitations, the obtained results should be interpreted as preliminary and indicative of the potential usefulness of the formulation in conditions associated with a weakened skin barrier.

## 5. Conclusions

As a result of the conducted study, a bio-mask prototype was developed, consisting of a multicomponent hybrid system based on an alginate hydrogel and lipid nanodispersion. The formulation is biodegradable, eco-friendly, and composed entirely of ingredients of natural origin. The bio-mask meets relevant safety requirements and complies with Regulation (EC) No 1223/2009 on cosmetic products.

Microbiological evaluations confirmed that the bio-mask is safe for use in human subjects. Instrumental studies conducted in five healthy volunteers indicate that the formulation increased skin hydration, reduced transepidermal water loss (TEWL) and erythema, improved skin elasticity, and decreased skin sensitivity. In vitro analyses showed that incorporation of silymarin and bakuchiol in the lipid nanodispersion enhanced the inhibition of collagen and elastin glycation compared to the reference hydrogel matrix. Additionally, the bio-mask promoted increased synthesis of type I procollagen, suggesting a beneficial effect on maintaining skin structural and functional integrity.

Overall, the obtained bio-hybrid system demonstrated moisturizing and skin-conditioning properties, as well as potential anti-aging effects. While these findings suggest its potential relevance in supportive skincare, including post-procedural applications, further studies involving compromised or post-treatment skin are required to confirm its effectiveness in such conditions.

## Figures and Tables

**Table 1:** The results of the microbiological purity tests of the bio-mask carried out following PN-EN ISO 17516:2014 [34].

Item	Name	Unit	Result	Under PN-EN ISO 17516:2014 [34]	Method
1.	Total number of mesophilic aerobic microorganisms (bacteria plus yeast and molds)	CFU/g	5.3 × 10^2^	≤1 × 10^3^ CFU/g or mL *	PN-EN ISO 21149:2017-07 [36] PN-EN ISO 16212:2017-08 [35]
2.	*Pseudomonas aeruginosa*	CFU/g	no presence in 1 g of the sample	not present in 1 g or 1 mL	PN-EN ISO 22717:2016-01 [39]
3.	*Staphylococcus aureus*	CFU/g	no presence in 1 g of the sample	not present in 1 g or 1 mL	PN-EN ISO 22718:2016-01 [40]
4.	*Escherichia coli*	CFU/g	no presence in 1 g of the sample	not present in 1 g or 1 mL	PN-EN ISO 21150:2016-01 [38]
5.	*Candida albicans*	CFU/g	no presence in 1 g of the sample	not present in 1 g or 1 mL	PN-EN ISO 18416:2016-01 [37]

* The limit is considered to be exceeded if > 2000 CFU/g or mL.

**Table 2:** Acceptance criterion for the neutralization level of preservatives.

Strain	Nvf *	Nvn **	Nv ***	Nvf ≥ 0.5 Nvn	Nvn ≈ Nv	Neutralization Effectiveness Demonstrated
*Pseudomonas aeruginosa* ATCC 9027	147	187	198	+	+	+
*Staphylococcus aureus* ATCC 6538	142	188	192	+	+	+
*Escherichia coli* ATCC 8739	140	185	190	+	+	+
*Candida albicans* ATCC 10231	138	181	188	+	+	+
*Aspergillus brasiliensis* ATCC 16404	47	84	88	+	+	+

* Nvf–number of CFU in 1 mL of the test sample (neutralizer + tested product), ** Nvn–number of CFU in 1 mL of the control sample (neutralizer + diluent), *** Nv–number of CFU in 1 mL inoculum control, +—acceptance criterion met.

**Table 3:** Reduction in the microorganism population introduced into the cosmetics expressed in logarithmic units.

Strain	Inoculum Density [CFU/g]	Contamination Level lgN_0_ **	lgRx *			lgR Requirements	lgR Requirements	Acceptance Criterion
			T7	T14	T28	Criterion A	Criterion B	
*Pseudomonas aeruginosa* ATCC 9027	5.5∙10^8^	6.74	2.98 (a)	4.38	5.74	T7 ≥ 3 T14 ≥ 3 and NI T28 ≥ 3 and NI	T7 not performed T14 ≥ 3 T28 ≥ 3 and NI	Met
*Staphylococcus aureus* ATCC 6538	5.2∙10^8^	6.72	2.53 (a)	3.41	5.72			Met
*Escherichia coli* ATCC 8739	5.4∙10^8^	6.73	2.62 (a)	2.69 (a)	5.73			Met
*Candida albicans* ATCC 10231	4.9∙10^7^	5.69	1.24	3.51	4.69	T7 ≥ 1 T14 ≥ 1 and NI T28 ≥ 1 and NI	T7 not performed T14 ≥ 1 T28 ≥ 1 and NI	Met
*Aspergillus brasiliensis* ATCC 16404	3.9∙10^7^	5.59	-	1.88	2.39	T14 ≥ 0 T28 ≥ 1	T14 ≥ 0 T28 ≥ 0 and NI	Met

* lgR_X_—reduction degree of the microorganisms, where R = lgN_0_ − lgN_X;_ ** lgN_0_—the number of microorganisms in 1 g of the sample immediately after contamination, expressed as a logarithm; lgN_X_—the number of microorganisms in 1 g of the sample after a specified time, expressed as a logarithm; T_7_, T_14_, T_28_—incubation period of 7, 14, and 28 days, respectively; N_I_—the number of microorganisms has not increased since the previous reading time; a—the permissible deviation from the acceptable value of the degree of reduction in the number of microorganisms is 0.5 log.

## Data Availability

The original contributions presented in this study are included in the article. Further inquiries can be directed to the corresponding author.

## Abbreviations

The following abbreviations are used in this manuscript:

AGEs	advanced glycation end products
CFU	colony-forming unit
DOE	statistical method of design of experiments
DSC	Differential scanning calorimetry
ECM	extracellular matrix
ELISA	enzyme-linked immunosorbent assay
FTIR	Fourier-transform infrared spectroscopy
H_0.25LN	hybrid hydrogel with lipid nanodispersion in a weight ratio 1:0.25
H_0.5LN	hybrid hydrogel with lipid nanodispersion in a weight ratio 1:0.5
H_1LN	hybrid hydrogel with lipid nanodispersion in a weight ratio 1:1
H_REF	hybrid hydrogel with lipid nanodispersion in a weight ratio 1:0
lgN0	the number of microorganisms in 1 g of the sample immediately after contamination, expressed as a logarithm
lgNX	the number of microorganisms in 1 g of the sample after a specified time, expressed as a logarithm
lgRX	reduction degree of the microorganisms
LN	lipid nanodispersion
NHDF cells	Normal Human Dermal Fibroblasts
NHEK	Normal Human Epidermal Keratinocytes
NI	number of microorganisms has not increased since the previous reading time
Nv	number of CFU in 1 mL inoculum control
Nvf	number of CFU in 1 mL of the test sample (neutralizer + tested product)
Nvn	number of CFU in 1 mL of the control sample (neutralizer + diluent)
PDA	Potato Dextrose Agar
SA	sodium salt of alginic acid from brown algae, medium viscosity
SA(S)	polymeric matrix contains sodium salt of alginic acid from brown algae and Spirulina
SDA	Sabouraud Dextrose Agar Medium
SEM	Scanning electron microscope
T7, T14, T28	incubation period of 7, 14, and 28 days, respectively
TEWL	Transepidermal Water Loss
TGA	Thermogravimetric analysis
TSA	Tryptic Soy Agar
